# Determinants of a successful problem list to support the implementation of the problem-oriented medical record according to recent literature

**DOI:** 10.1186/s12911-016-0341-0

**Published:** 2016-08-02

**Authors:** Sereh M. J. Simons, Felix H. J. M. Cillessen, Jan A. Hazelzet

**Affiliations:** 1 Department of Public Health, Erasmus University Medical Center, Rotterdam, The Netherlands; 2 Department of Primary and Community Care, Radboud University Medical Center, Nijmegen, The Netherlands

**Keywords:** Problem-oriented medical record, Electronic Health Record, Review, Implementation

## Abstract

**Background:**

A problem-oriented approach is one of the possibilities to organize a medical record. The problem-oriented medical record (POMR) - a structured organization of patient information per presented medical problem- was introduced at the end of the sixties by Dr. Lawrence Weed to aid dealing with the multiplicity of patient problems. The problem list as a precondition is the centerpiece of the problem-oriented medical record (POMR) also called problem-oriented record (POR).

Prior to the digital era, paper records presented a flat list of medical problems to the healthcare professional without the features that are possible with current technology. In modern EHRs a POMR based on a structured problem list can be used for clinical decision support, registries, order management, population health, and potentially other innovative functionality in the future, thereby providing a new incentive to the implementation and use of the POMR.

**Methods:**

On both 12 May 2014 and 1 June 2015 a systematic literature search was conducted. From the retrieved articles statements regarding the POMR and related to successful or non-successful implementation, were categorized. Generic determinants were extracted from these statements.

**Results:**

In this research 38 articles were included. The literature analysis led to 12 generic determinants: clinical practice/reasoning, complete and accurate problem list, data structure/content, efficiency, functionality, interoperability, multi-disciplinary, overview of patient information, quality of care, system support, training of staff, and usability.

**Conclusions:**

Two main subjects can be distinguished in the determinants: the system that the problem list and POMR is integrated in and the organization using that system. The combination of the two requires a sociotechnical approach and both are equally important for successful implementation of a POMR. All the determinants have to be taken into account, but the weight given to each of the determinants depends on the organizationusing the problem list or POMR.

**Electronic supplementary material:**

The online version of this article (doi:10.1186/s12911-016-0341-0) contains supplementary material, which is available to authorized users.

## Background

Within medical care a patient’s medical record is the main source of patient information. How this medical record is organized varies. A problem-oriented approach is one way of organizing a medical record. Nowadays a medical record is mostly an Electronic Health Record (EHR).

One of the core elements of an EHR (Electronic Health Record) is the problem list. The problem list can be defined as “a list of current and active diagnoses as well as past diagnoses relevant to the current care of the patient” [1]. Opinions differ on what problems should be on the problem list. Therefore it is important to define a policy for maintaining the problem list but such a policy is often hard to implement [2]. The problem list is the centerpiece of the problem-oriented medical record (POMR), also called problem-oriented record (POR). The POMR – a structured organization of patient information per presented medical problem [3] – was described at the end of the sixties by Dr. Lawrence Weed [4] to aid dealing with the multiplicity of patient problems. Observations, assessments and healthcare plans are grouped by patient problem. Writing progress notes in the subjective-objective-assessment-plan (SOAP) format can structure the data even further.

There is no clear or widely supported standard in the problem-oriented way of working. Therefore implementation can be difficult. Besides the lack of a standard there are further limitations to the POMR and many modern EHR systems lack functionality. This was already described in the nineties [5]. However the POMR fits in the trend of care becoming more patient-centered. A problem-oriented approach is also useful because patients can relate easily to the problems on the problem list and assess and respond to them [6]. In the Netherlands the intention is to come to a nationwide “model” for a POMR. To learn from previous initiatives and implementation projects where the POMR and/or problem list played an important role we conducted this systematic literature review. Prior to the digital era, paper records presented a flat list of medical problems to the healthcare professional without the features that are possible with current technology. In modern EHRs structured problem lists can be used for clinical decision support, registries, linking data to problems, order management, population health, and potentially other innovative functionality in the future, thereby providing a new incentive to the implementation and use of the POMR. The success of these new uses of the POMR will depend, in addition to many other variables, on the EHR functionality and how it is implemented.

Here, we present a systematic review to identify the determinants that influence the implementation and use of a problem-oriented approach in EHRs.

The objective of this study is to extract determinants of the implementation and use of problem list to support the POMR from recent literature, and thereby support institutions in a successful implementation and use of the POMR.

## Methods

### Collecting literature

A literature search was conducted on 12 May 2014 in Embase.com, Medline (OvidSP), Web of Science, Cochrane, PubMed publisher and Google Scholar to find articles from which determinants for the successful implementation and use of a problem list to support the POMR could be extracted. The basic search string was made in Embase.com, the search strings for the other databases were derived from the Embase.com search string. On 1 June 2015 the search was repeated in the databases Embase.com, Medline (OvidSP), Web of Science, Scopus, PsychINFO, Cinahl, Cochrane, PubMed publisher and Google Scholar.

The final search string used in Embase.com was: ((‘medical record’/exp OR ‘medical documentation’/de OR (((medical* OR electronic* OR patient* OR health* OR dental* OR personal* OR hospital* OR nurs* OR psychiatr* OR computer*) NEAR/3 (record* OR documentat*)) OR ehr OR emr OR ehrs OR emrs):ab, ti) AND ((problem* NEAR/3 (‘oriented’ OR ‘orientated’ OR ‘orientation’))):ab, ti OR (CPOMR OR POMR OR (problem-oriented NEXT/3 (record* OR approach* OR system* OR report* OR method*)) OR (problem NEXT/3 (list OR lists OR summar*)) OR problemlist*):ab, ti).

The final search strings for the other databases can be found in the Additional file 1.

### Literature review

Articles were included in two stages. One reviewer (SMJS) made the first selection based on title and abstract, then two reviewers (SMJS, FC) made the selection on full text by first reviewing the full texts individually and secondly discussing all of the individually included articles in face-to-face meetings. If the two reviewers did not agree on a specific inclusion/exclusion of an article, JH made the final decision.

#### Inclusion/exclusion criteria

We chose a ten-year period and excluded articles published prior to 2004 because we wanted to focus on recent developments in the use of the POMR. Saturation of determinants was expected to occur within the selected ten-year period. We therefore started both the inclusion/exclusion of articles and the literature analysis with the most recently published articles. The literature analysis was conducted simultaneously with the full text review.

From the retrieved abstracts SMJS removed all articles not written in English, all articles using animal models and all conference abstracts. SMJS then removed articles the title and abstract of which showed that the article did not include some sort of medical record and problem or problem list. When such exclusion could not be certain from the title and abstract the full text was analyzed. These criteria are also summarized in Table 1.

**Table 1:** Summary of inclusion/exclusion criteria used on articles in the two rounds of selection, abstract and full-text based

Inclusion criteria	Exclusion criteria
Title-abstract	
Written in English	Non-Human
Use of a problem list or orientation within medical records	Conference abstracts
Hospital or clinic setting	Published before 2004
About clinicians or patients	
Patients as subject of care	
Additional for full texts	
Main subject: the use of the medical record	Published before 2011

In the full text review we (SMJS and FC) excluded articles focusing only on a technical description of POMR models as technical models are intrinsically related to vendors or specific software. Also, articles were excluded the main subject of which was not the POMR or in which the POMR was merely mentioned as a way to organize medical records without focusing on the use or implementation of the POMR. Thirdly, articles published in 2010 and before 2010 were excluded. This is because the literature analysis showed a saturation of determinants in articles published in 2012 and neither were further determinants found in 2011.

### Literature analysis

The literature analysis was conducted, starting with the most recent articles until saturation of determinants occurred. In order to extract the determinants SMJS first selected quotes which made a statement about the POMR. After that the quotes were categorized by content. These categorized quotes led to generic determinants, independent of specific vendor or system. FC and JH agreed on the categorization of the quotes and the determinants formulated.

## Results

The systematic literature search of 12 May 2014 resulted in 3338 unique articles. Of these 3338 articles SMJS included 297 based on title and abstract. The full text review of these 297 articles led to the inclusion of 33 articles. The literature analysis of these 33 articles showed a saturation of determinants to a total of 12 (see Table 2) in articles published between 2012 and 2014. Articles published in 2011 were then added to the literature analysis to make sure that none of the determinants was overlooked. Articles published in 2011 did not result in extra determinants. Therefore all articles published in 2010 and before were excluded. During the literature analysis we excluded one more article, as the main subject of the article proved not to be about the use of the medical record after all.

**Table 2:** Determinants with definition and extracted statements of importance

Determinant- definition	Relevant Statements
Clinical practice/reasoning Clinical practice and or reasoning used we defined as the normal clinical routine of a clinician.	• The problem list can support the clinical practice ○ by giving up-to-date information about a patient enabling management of important health factors [7, 9] ○ by providing a guideline to secure all problems are discussed clinically and with the patient [12] ○ by sorting information, source oriented and in chronological order [8, 12] ○ by enabling clinical decision support [15, 16] ○ when notes are efficient and to the point [2, 16] • It should be fully integrated in the workflow of a patient visit otherwise the use of the POMR will only increase work load rather than increasing efficiency and quality of care [11, 12] • It should be possible to merge or link problems and their interventions, as problems are not always treated one at the time, to prevent fragmentation of patients data [8, 10, 12, 13] • The mandatory items should be useful and applicable to the specific care situation, for diagnoses and interventions it makes sense to have mandatory items, but for routine interventions these should not be mandatory [8] • When supporting clinical practice it is important to remember that attitude towards the problem list and its use can differ substantially between clinicians. Therefore different ways of working with the problem list should be supported [14, 17].
Complete and accurate problem list How to deal with the wish of complete and accurate problem list.	• Participation of the patient in reviewing the problem list allows the clinician to update the problem list accordingly and can support a meaningful patient-clinician dialogue [6, 11] • Problem list must be maintained and updated according to organization-wide guidelines, in order to be reliable and to give a relevant overview of available patient information [2, 7, 9, 12, 14, 15, 17–20, 23, 24, 27–30] • All caregivers should be able to update the problem list, organizations should make, a choice if there should be separate lists for different caregivers [14, 18] • System support can be used to complete the problem list [14, 15, 20, 21, 25, 26, 31] • Clinical notes should be linked to problems [13, 15, 22, 29] • Reviews of the problem list together with patients can improve the quality of the list [6, 11, 15]
Data structure/content Data structure and content is about the structure of the data and the content of the record or problem list.	• There are a lot of different opinions about what, diagnosis, complaints, concerns, interventions, should be added to the problem list and who has to add this. Policy is needed so that users use the problem list in the same and proposed manner [9, 12–14, 19] • It should be possible to link problems, so relationships between them can be made clear, and similarly it should be possible to link clinical notes to multiple problems [8, 10, 13]. • Problem list should have a dictionary/taxonomy behind them so codes from codesytems like ICD 9 ICD 10 and SNOMED, can be extracted. [6, 11, 18, 22] • The required and mandatory information should make intuitive sense to the user. For example, enforcing information entry for routine interventions is not necessarily useful [8] • It has to be possible to specify coded entries further by providing free text at entry [12] • All clinicians should be able to register their problems with the appropriate coded problem list [12, 22]
Efficiency Efficiency is gaining the most out of your time and resources.	• The POMR will only be adopted successfully if it is time efficient ○ The amount of work to fill in the format should be equivalent or reduced compared to currently employed use of POMR [2, 8, 32]. ○ Clinical notes should be to the point and optimized [2, 8, 16] ○ The time gain should result from providing a quick overview of the patient [7, 14] ○ Electronic POMR should be fully integrated in the workflow of a patient visit otherwise it will increase work load [11, 12] ○ System support should help to improve efficiency (see below) • The quick overview of the patient is important ○ to ensure increased efficiency (as outlined above). ○ to provide, efficient and therefore high quality care [7, 14] • Using encoded problem list items ○ Makes sure all professionals using the list agree on the meaning of an element [22] ○ Makes clinical descision support possible [22], see also below.
Functionality Functionality is used in this article for technical functions/features the system should have.	• It has to be possible to link problems and interventions. Moreover when updating the original input of the problem list it should be possible to change the hierarchy of the problem list when the diagnosis becomes more precise [8, 10, 12, 13, 27, 35, 36] • A search function has to be implemented to prevent redundant entries [18, 35] • The encoded list should be able to handle synonyms and free text entries or misspelled entries and should have an auto-suggestion feature [2, 6, 12, 31, 32] • Filter and custom views should be possible [8, 12, 18, 24] • Clinical decision support should check the problem list and assist the clinician in filling out the problem list [15, 21, 25, 26, 33–35, 37] • It has to be possible to specify coded entries further by providing free text at entry [12] • The problem list should work as a table of contents of the medical record [13, 18]
Interoperability Interoperability is the possibility to transfer data between systems without losing its information and context.	• The problem list should be filled from all available clinical information systems to provide a complete view on the patient and not loose data entered in another system [2, 18, 28, 37] • Problem list should have a dictionary/taxonomy behind them so codes from code systems, like ICD 9 ICD 10 and SNOMED, can be extracted, enlarging the interoperability [6, 11, 18, 22]
Multi-disciplinary The definition used in this article for multi-disciplinary is the combination of different clinicians and health professionals.	• The problem list should support communication between disciplines and coordinate the care of the patients’ problem [7, 10, 18, 22, 36, 37] • An agreed approach and management support is necessary to maintain a multi- disciplinary problem list [14, 17, 22] • Keeping the problem list up to date and accurate is important when working across disciplines on the same patient [19, 21] ○ The more clinicians can and are allowed to add to the problem list, the more complex the list becomes to maintain [18] ▪ When disciplines are responsible for updating the problem list entries of their own expertise this could be solved [2, 22] • Standardized terminology enlarges understanding between clinicians [18, 22] • Clear guidelines and instructions on usage will help (paramedical) professionals to know what to include on the lists. Moreover the view of other clinicians of the patients’ problems can be of great importance in the care of patients [12, 14, 22, 23, 27]
Overview of patient information In an electronic health record overview of the patient data is of vital importance.	• In an EHR, providing overview of the patient data is of vital importance [8, 27] • It should be possible to link problems and interventions, as problems are not always treated one at the time, to prevent fragmentation of the patients data [8, 10, 12, 13] • The problem list should represent the patient data in a coherent and logical order, so it provides a cornerstone of the EHR, preventing errors due to missing information [11, 15, 18, 21, 25, 36] • If well maintained and structured, a problem list can assist a multi-disciplinary approach [10, 13, 16, 36] • Policy should help with constructing the overview of patient data, providing guidelines for adding or leaving problems of the list reducing confusion and preventing missing information [14]
Quality of care The definition of quality of care is used in the broadest sense of the term, all which can influence the care of the patient.	• If all patient information is related to problems and the problem list is often updated it allows for an evaluation of the efficacy of the treatment [7, 13, 15, 22, 33] • Communication and coordination between health professionals is supported by the problem list [7, 10, 15, 18, 21, 22] • Clear policy on what to put on the problem list and for the users clear structure is essential [9, 10, 14, 18, 19, 27, 29] • The problem list is a valuable tool to get overview of the data of (unfamiliar) patients [7, 8, 11, 12, 14, 15, 18, 25, 27] • The length of the problem list indicates the complexity of the patient [12] • The problem list helps practitioners to identify the most important health factors for each patient, enabling personalized care [9, 11, 31] • All clinicians should be able to update the problems on the problem list, collegues can review the problem list and improve patient care by keeping it updated [14, 22, 36, 37] • When patients are able to review their own problem list, this allows them to direct improve their own care and health [6]
System support Systems can support the user of the POMR in many ways; Clinical decision support, autosuggest etc.	• Prescribing medication, and ordering diagnostics or interventions can automatically populate the problem list. Important with such features is that the system checks if the problem is already on the list, to avoid redundancy of problems on the list. [15, 21, 25, 26, 33–35, 37]. • Systems can be configured in a way that they can detect omissions in the problem list to give clinicians the opportunity to correct it accordingly (also in clinical decision support). The user should always be the one to accept or authorize the problem to the list, automatic adding of problems is not desirable [16, 18, 20, 21, 25, 26] • Clinicians are more likely to contribute to the problem list if the system supports them with meaningful triggers [12] • With consistency across the problem list and encoded entries the system can support reusing the information for multiple purposes, for instance billing and quality assessments and also the other way around: from billing codes to problems, for example [11, 14, 16, 25, 26, 37]. • Natural language processing, from free text extracting codes, can help populate and complete the problem list. Language in which this feature is developed and the completeness of the processing has to be taken into account before using this feature [15, 29, 38] • If diagnoses have to be registered in another module they should automatically be part of the problem list [2, 18, 28, 37] • The system should be able to make comprehensive summaries of the record using the problem list [39] • The system should present related terms to the user when entering problems [31]
Training of Staff With training of staff the professional training is meant, how many years of experience and the training on using the system.	• Users should be trained in using the problem list, its features and policies on the problem list. This increases the correct use of the problem list [12, 14, 25, 40, 42] • Experience in medicine and medical profession also influences the use of the problem list this should be taken into account in training of staff [17, 25, 41] • Training can increase the correct use of the problem list [12, 14, 25, 40, 42]
Usability The definition of usability in this article is user-friendliness and user interface.	• Custom views and filters should be possible [2, 8, 18, 39] • The interface should be intuitive and efficient [8, 13, 39] • The encoded dictionary should be able to identify synonyms, misspelled entries and handle free text [2, 6, 12, 31, 32] • System support has to be integrated at logical moments in the notes registry (e.g. orders and medication) [17, 25] e.g. actionable items • The clinical status of the problems should be easily identifiable [18]

On 1 June 2015 the systematic literature search was repeated. It resulted in 4298 unique articles unique in that search. The selection procedure used in the 12 May 2014 search was repeated and led to the inclusion of 5 more articles. None of these articles were found in any of the databases that were added in the 2015 search. This repeated search did not result in any more determinants. The total result of both searches was 38 included articles: 11 from 2011, 7 from 2012, 12 from 2013 and 8 from 2014.

These 38 articles led to 12 generic determinants. In Table 2 these determinants are shown in alphabetical order. In general determinants are rarely perfectly distinct and show considerable overlap. This is also the case with our determinants. For example, usability and functionality cannot be distinguished completely as they are interdependent. Still, the distinction is big enough for these determinants to both be relevant. Our determinants are defined in more detail by relevant statements relating to the determinants in the articles.

## Discussion

The POMR is patient centered and enables clinicians to have a holistic and integral view of the patient. The POMR can give a quick overview of the most important current and past medical issues the patient has. So far there is no international standardized way of working for a POMR or the use of a problem list. Therefore there is a great variety of the use of the problem list within medical practice from no use at all to hospital wide use. Using POMR or even just the problem list on paper is not an easy task to accomplish and can be very cumbersome. Nowadays, computers and other electronic devices are used in every aspect of the medical practice. This technology could be a turning point in the use and the rediscovery of the clinical relevance of the POMR. Our systematic review is therefore timely indicating which determinants play a role in the use, design and implementation of the electronic POMR. From the literature we distilled 12 determinants that influence the use and implementation of the problem list to support the POMR. The determinants are all vendor independent. When these determinants are given adequate attention, they can contribute to a successful implementation of the POMR.

### Clinical reasoning [2, 7–17]

The POMR supports daily clinical practice and clinical reasoning. The Problem list should be the integrating part of the medical record. All data such as observations, assessments and plans (diagnostics, treatments and information to patients) must be associated with a problem on the problem list. Furthermore, all data must be easily retrievable with the option for different views depending on the context.

### Complete and accurate problem list [2, 6, 7, 9, 11–15, 17–31]

It is important that healthcare professionals can rely on the content of the problem list. Starting with clear and agreed upon guidelines stating what is a problem to put on the list and how to keep the status of the problem up-to-date. The multi-disciplinary use is an important issue that should be covered by guidelines. These guidelines should be made within the organization the POMR is implemented in, as it is important to match the use of POMR to the size, complexity and previous situation of the organization.

### Data structure/content [6, 8–14, 18, 19, 22]

Coded entries to the problem list can attribute to semantic interoperability. Various terminology systems can be used, also in combination, but internationally SNOMED CT is to be preferred. Free text should be an option to indicate that there is a problem when it comes to a hard or impossible observation to code. Also nuances, context or clinical status such as ruled in or ruled out can be of crucial importance. Forced coding without the possibility to add free text can make definitive diagnosis imprecise.

### Efficiency [2, 7, 8, 11, 12, 14, 16, 22, 32]

One of the main concerns of clinicians is that new systems require a different way of working. Functionality integration into clinical practice, training of the clinicians, accurate and up-to-date problem lists, and system support can all increase the efficiency and effectiveness of the use of the POMR.

### Functionality [2, 6, 8, 10, 12, 13, 15, 18, 21, 24–27, 31–37]

The EHR is an enabler for the POMR in the computer era. Linking problems, interventions, notes, supporting the clinicians with the huge amount of data in EHR’s, context depending views of all data, functions for maintainability i.e. scan function to avoid redundancy may be presented to support the clinicians. Systems should be able to handle free text and misspelled entries with an autosuggest function so coded lists will be used more and more efficient. Clinical decision support is one of the other key functionalities why the digital POMR could succeed. With the enormous growth of knowledge, it is impossible to know everything.

### Interoperability [2, 6, 11, 18, 22, 28, 37]

Closely related to data structure/content, efficiency and accuracy. The problem list is an important communication tool. Problems should be recorded unambiguously and only once to prevent miscommunication and redundancy.

### Multi-disciplinary [2, 7, 10, 12, 14, 17–19, 21–23, 27, 36, 37]

With all medical disciplines working together with the patient at the center the problem list should also be multi-disciplinary. A multi-disciplinary list creates a holistic view of the patient and supports patient-centered care. Clear guidelines for the use of the problem list are essential especially in a multidisciplinary setting. The problem list can be used to coordinate care and communicate effectively about the patient among the care team but also with the patient. Use of standardized language is a key element of multi-disciplinary work and problem list use.

### Overview of patient information [8, 10–16, 18, 21, 25, 27, 36]

Is of vital importance to be quickly and fully informed about what is going on with the patient, especially as many clinicians work under time pressure in daily clinical practice. The problem list should support overview and not increase fragmentation of patient information. Linking data to one or more problems contributes to overview. Filters are important such as chronologically, source and problem oriented. The problem list can function as an index to the patient medical record.

### Quality of care [6–15, 18, 19, 21, 22, 25, 27, 29, 31, 33, 36, 37]

As quality of life for the subject of care i.e., the patient is the main goal for health care providers. The POMR is an enabler to improve quality of care. It enables clinicians to organize and structure data. Problems can be viewed one by one but also in a more multi-disciplinary holistic way. The POMR will also contribute to transparency and accountability. Viewing possibilities and even corrective options for the patient will also contribute to higher quality of care.

### System support [2, 11, 12, 14–16, 18, 20, 21, 25, 26, 28, 29, 31, 33–35, 37–39]

As an assistant to give suggestions for diagnosis or treatment as a form of clinical decision support. This is a helpful feature to increase the use of the problem list, but the system should not automatically add problems without the interference of a clinician. Autosuggest and related terms can increase the efficiency of the use of the coded lists. When clinicians experience those features they would be more prone to use coded problem lists. Natural language processing could be helpful, although it depends on how far this is developed and the language in which the NLP is developed.

### Training of staff [12, 14, 17, 25, 40–42]

Both experience in medicine as experience in EHR and problem lists, increase the correct use of the list. When implementing the POMR, make sure proper training is given and training is maintained throughout the years using the POMR. Without proper training, monitoring and feedback clinicians can simply fall back to old habits and use the list incorrectly. Incorrect use of the problem list undermines the reliability of the list and therefore clinicians will use the problem list less. This can become a vicious circle.

### Usability [2, 6, 8, 12, 13, 17, 18, 25, 31, 32, 39]

Besides functionality usability is of utmost importance. Functions of a system can be very advanced and extensive but if the user interface is not intuitive and easy to use and without useful and fast feedback it will be time consuming with the risk that people do not use it. Encoded libraries should be fast and be able to identify synonyms and misspelled entries. The use of best practice advice options can improve the usability substantially.

Looking at the determinants we can distinguish two main subjects: the system in which the POMR is integrated and the organization with its users in which the system is used. Both the system and the organization should be well prepared for using the POMR. Functionality, usability, system support, overview of patient information, interoperability, supporting clinical reasoning and data structure/content can be designed, naturally while designing the system the maturity of the organization and associated policies has to be taken into account. The organization needs to be ready and willing to use the POMR. More specifically, willing to integrate the problem list into daily routine, using it multi-disciplinary, discussing it with the patient, be sufficiently trained and follow the guidelines on using the problem list. This requires a sociotechnical approach and is equally important for successful implementing a POMR. All the determinants have to be taken into account, but the weight given to each of the determinants depends on the organization the POMR and problem list is used and implemented in. Therefore we did not distinguish important and slightly less important determinants.

## Conclusion

This study shows that there is more to a successful implementation of a problem list to support the POMR than the technical software and hardware associated with electronic patient records. Both the technical and organizational aspects should be taken into account while implementing a problem list or POMR. The statements defining our determinants in more detail can be of use here.

The successful implementation and use of a problem list to support the POMR are related to 12 vendor independent determinants. All these determinants taken into account together appear to be a critical success factor for a successful implementation. We have not assigned weight to our determinants as their relative importance depends on various factors and should be determined within the organization the implementation is planned for.

### Limitations

This research has its limitations. Firstly, we wanted to focus on the latest insights and performed a systematic literature search, and analyzed the articles of the last five years. As saturation occurred within these five years we are confident that we managed to gain the latest insight in the use of a problem list and the POMR. Still, there is always a chance of there having been relevant older articles.

Secondly, as some determinants are closely related, describing them separately may come across as somewhat unnatural. Despite these limitations the results of this systematic review can contribute to a better implementation and use of a problem list and the POMR.

### Further research

Further research is required to establish the predictive quality of our determinants. Studies of the use of a problem list to support the POMR in daily clinical practice over several years are rare, especially generalizable studies combining qualitative and quantitative evaluation. Also the linking of data to items on a problem list is hardly scientifically described. Further research needs to focus on the use of the POMR and the determinants. It would be advisable to develop a scorecard to objectify the relevance of a determinant in a successful implementation of the POMR.

## Abbreviations

EHR, electronic health record; POMR, problem-oriented medical record; SNOMED, systematized nomenclature of medicine; ICD 9–10, international classification of diseases and related health problems

## Additional file

Additional file 1: Supplement data POMR simons. The file contains a Prisma flow chart and additional search strings used to collect the articles. (DOCX 42 kb)
