# Supplementary material for: Determinants of a successful problem list to support the implementation of the problem-oriented medical record according to recent literature
Source: BMC Med Inform Decis Mak. 2016 Aug 2;16:102. doi: 10.1186/s12911-016-0341-0 (PMC4970280; doi:10.1186/s12911-016-0341-0)
Supplement: Additional file 1: — Supplement data POMR simons. The file contains a Prisma flow chart and additional search strings used to collect the articles. (DOCX 42 kb) [file 12911_2016_341_MOESM1_ESM.docx]

# Supplement data

## PRISMA flow diagram

#
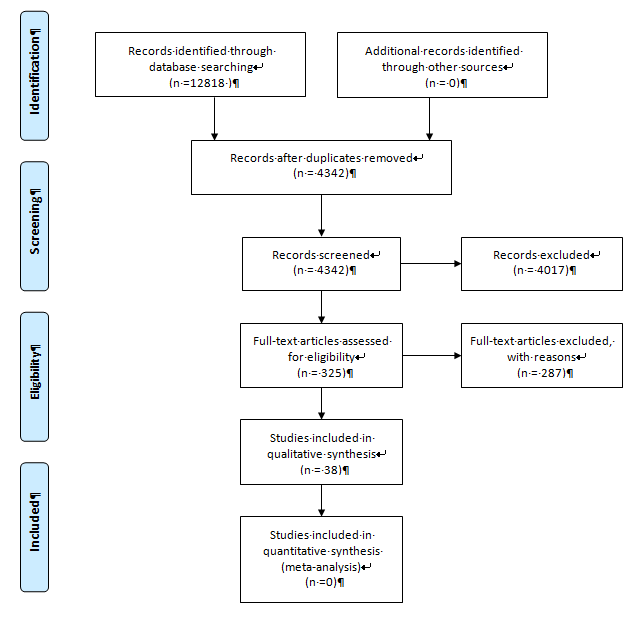


Figure 1 PRISMA diagram of the conducted systematic review [[41](#_ENREF_41)]

## Searches in other data bases

**Medline (OvidSP) 2544**

((exp "medical records"/ OR (((medical* OR electronic* OR patient* OR health* OR dental* OR personal* OR hospital* OR nurs* OR psychiatr* OR computer*) ADJ3 (record* OR documentat*)) OR ehr OR emr OR ehrs OR emrs).ab,ti.) AND ((problem* ADJ3 (orient*))).ab,ti. OR "Medical Records, Problem-Oriented"/ OR (CPOMR OR POMR OR (problem-oriented ADJ3 (record* OR approach* OR system* OR report* OR method*)) OR (problem ADJ3 (list OR lists OR summar*)) OR problemlist*).ab,ti.)

**Cochrane 44**

(((((medical* OR electronic* OR patient* OR health* OR dental* OR personal* OR hospital* OR nurs* OR psychiatr* OR computer*) NEAR/3 (record* OR documentat*)) OR ehr OR emr OR ehrs OR emrs):ab,ti) AND ((problem* NEAR/3 (orient*))):ab,ti OR (CPOMR OR POMR OR (problem-oriented NEXT/3 (record* OR approach* OR system* OR report* OR method*)) OR (problem NEXT/3 (list OR lists OR summar*)) OR problemlist*):ab,ti)

**Web-of-science 201**

TS=((((((medical* OR electronic* OR patient* OR health* OR dental* OR personal* OR hospital* OR nurs* OR psychiatr* OR computer*) NEAR/3 (record* OR documentat*)) OR ehr OR emr OR ehrs OR emrs)) AND ((problem* NEAR/3 (orient*))) OR (CPOMR OR POMR)))

**PubMed publisher 17**

(((medical record*[tiab] OR electronic record*[tiab] OR patient record*[tiab] OR health record*[tiab] OR dental record*[tiab] OR personal record*[tiab] OR hospital record*[tiab] OR nursing record*[tiab] OR psychiatric record*[tiab]) OR ehr[tiab] OR emr[tiab] OR ehrs[tiab] OR emrs[tiab])) AND ((problem orient*[tiab]))) OR (CPOMR[tiab] OR POMR[tiab] OR ("problem oriented"[tiab] AND (record*[tiab] OR approach*[tiab] OR system*[tiab] OR report*[tiab])) OR problem list*[tiab] OR problem summary list*[tiab] OR problemlist*[tiab])) AND publisher[sb]

**Google Scholar**

"medical|electronic|patient|health|dental|personal|hospital|nursing|psychiatric record|records"|ehr|emr|ehrs|emrs problem oriented|orientated|orientation"|CPOMR|POMR

|  |
| --- |
